# Metal artifacts in intraoperative O-arm CBCT scans

**DOI:** 10.1186/s12880-020-00538-4

**Published:** 2021-01-06

**Authors:** Juha I. Peltonen, Touko Kaasalainen, Mika Kortesniemi

**Affiliations:** HUS Medical Imaging Center, Radiology, University of Helsinki and Helsinki University Hospital, P.O. Box 340, 00029 Helsinki, Finland

**Keywords:** Cone-beam computed tomography, Image quality, Metal artifacts

## Abstract

**Background:**

Cone-beam computed tomography (CBCT) has become an increasingly important medical imaging modality in orthopedic operating rooms. Metal implants and related image artifacts create challenges for image quality optimization in CBCT. The purpose of this study was to develop a robust and quantitative method for the comprehensive determination of metal artifacts in novel CBCT applications.

**Methods:**

The image quality of an O-arm CBCT device was assessed with an anthropomorphic pelvis phantom in the presence of metal implants. Three different kilovoltage and two different exposure settings were used to scan the phantom both with and without the presence of metal rods.

**Results:**

The amount of metal artifact was related to the applied CBCT imaging protocol parameters. The size of the artifact was moderate with all imaging settings. The highest applied kilovoltage and exposure level distinctly increased artifact severity.

**Conclusions:**

The developed method offers a practical and robust way to quantify metal artifacts in CBCT. Changes in imaging parameters may have nonlinear effects on image quality which are not anticipated based on physics.

## Background

An evolving three-dimensional (3D) imaging technique, called cone-beam computed tomography (CBCT), has gained increasing interest and usage in medical imaging. This versatile imaging method is used for diagnosis and treatment planning purposes for example in dentistry, orthopedics, neurosurgery, and interventional radiology. Furthermore, the application of CBCT systems is increasing in operating rooms where it is used for intraoperative 3D imaging. Several publications have shown that 3D image guidance may provide significant improvements for minimally invasive and more accurate instrument placement in various surgeries [1–6]. Thus, CBCT-based image guidance has the potential to reduce complication rates and improve cost-effectiveness [7]. Similar to other novel techniques, it involves a clear learning curve for surgeons performing the image-guided procedures [8, 9].

Despite the proven benefits, increased use of intraoperative CBCT imaging has also raised concerns about the harmful effects of ionizing radiation (e.g., radiation-induced cancer). Specifically, the introduction of an O-arm imaging system, a dedicated 2D/3D surgical imaging platform designed for orthopedic and neurological surgery, is prone to increase the radiation dose due to the rotational exposure included as a general step in all CBCT imaging. A wide range of applicable image quality levels is available in O-arm imaging, leading to a large variation in patient doses. For example, the effective dose in a spinal surgery with an O-arm system has been reported as being between 0.6 and 13 mSv, depending on the selected imaging protocol and patient characteristics [10–12]. It has also been claimed that the radiation doses resulting from the spinal surgery with an O-arm device can be reduced 5–13 times compared with the manufacturer’s default settings without a negative impact on the required image quality [13].

Within the radiological optimization process, it is essential to verify that the image quality in the intraoperative CBCT scans is adequate for the reliable perception of target structures. Image quality may deteriorate significantly due to metal implants and prosthesis whenever these objects are present due to previous operations [11, 14–16]. The high X-ray attenuation of metal causes photon starvation and beam hardening artifacts in the images [17]. The artifacts may hamper the visibility of objects of interest and affect the reliable clinical evaluation of, for example, implanted pedicle screws. However, there are various methods of suppressing these artifacts [18]. Furthermore, the careful acquisition strategies, good clinical workflow and appropriate anatomical placement of the scan field of view (FOV) are important factors while pursuing optimal image quality [14].

The aim of this study was to develop a robust method to quantify metal artifacts in CBCT images. The method was applied to O-arm CBCT scans of an anthropomorphic phantom with metal inserts and by using varying imaging parameters.

## Methods

### O-arm CBCT device

The O-arm (Medtronic Inc., Louisville, CO, USA) is a mobile intraoperative CBCT system designed for both 2D and 3D imaging. The system used is a second-generation O-arm based on a conventional x-ray tube and a 40 cm × 30 cm flat panel detector. In 3D mode, the system enables 360-degree rotation with a rotation time of 13 s in the standard operating mode. In addition to the predefined imaging protocols, a user can manually select the tube voltage (kVp) and exposure (mAs; a product of the tube current and irradiation time per rotation) used for imaging. The system can also be used jointly with an additional navigator system to perform more accurate and safer image-guided operations [4, 11, 19, 20].

### Image acquisition

The pelvic part of an anthropomorphic phantom (ATOM Model 702-D, CIRS, Norfolk, VA, USA) was used in the study to mimic an orthopedic patient. The phantom accurately represents patient sizes from teenage pediatric patients to small adults. The radiodensity of the different structures inside the phantom correspond to the respective tissues in the human body.

**Fig. 1:**
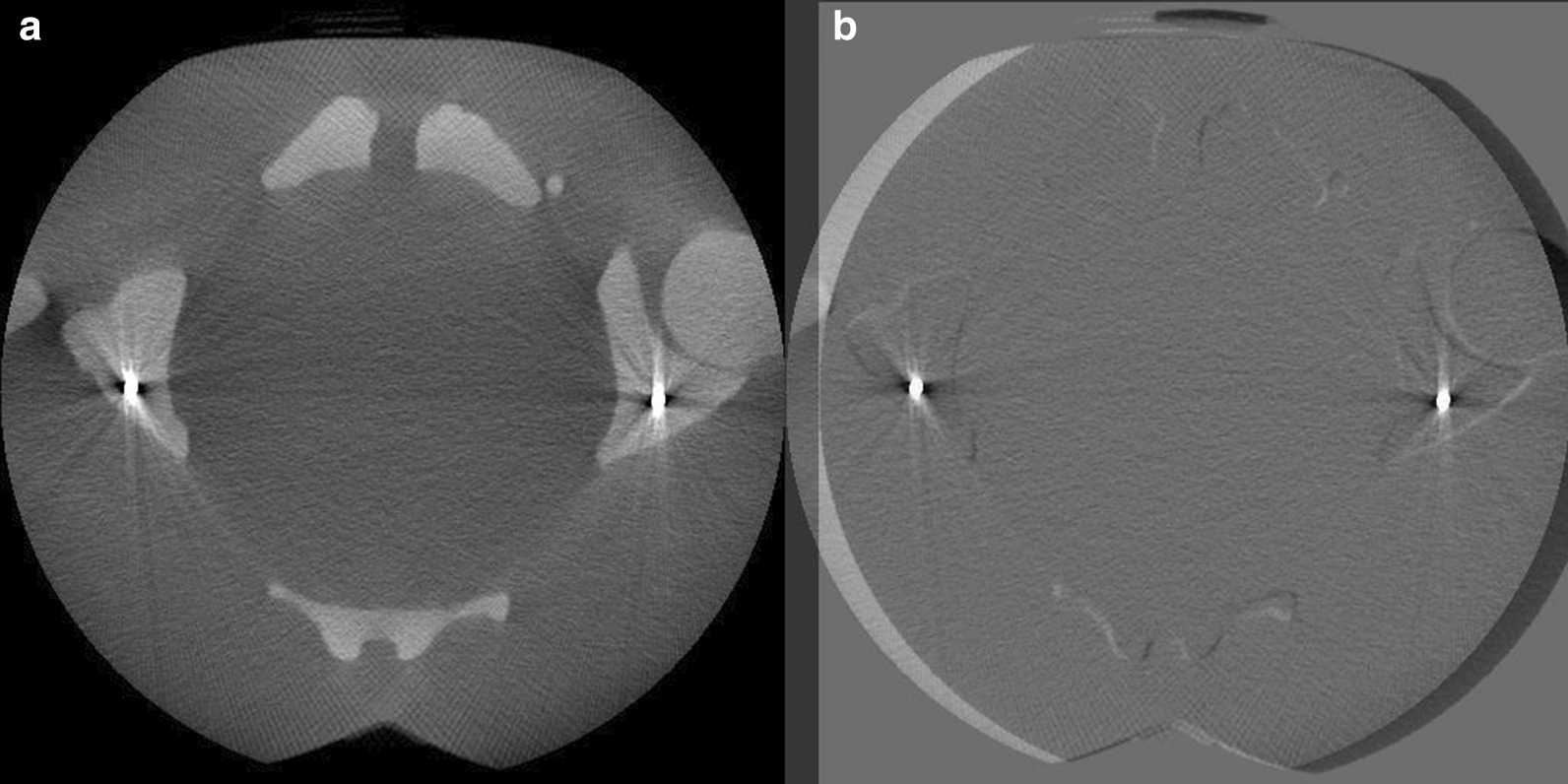
**a** Example of image artifacts induced by the metal rods in the axial image slice. **b** The same image slice as in a) subtracted with the slice from the artifact-free imaging volume

The O-arm device was used in the scanning with the imaging protocol settings presented in Table 1. The corresponding effective dose in each protocol was calculated with the rotational version of the PCXMC 2.0 Monte Carlo dose simulation program (Radiation and Nuclear Safety Authority, STUK, Finland). The dose simulation was performed according to corresponding CBCT scan parameters and equipment characteristics including exposure geometry, x-ray tube filtration, tube voltages, tube current, scan FOV, and anatomical position of the scan. The air kerma in the CBCT scan isocenter corresponding to each imaging protocol was measured according to IAEA and IEC recommended method for wide beam dosimetry [21, 22]. The measured air kerma values were used to scale the simulation results according to the applied mAs in each CBCT scan. All protocols included scanning both with and without metal inserts. Also, the laterality of the metal inserts and the isocenter position in the phantom were studied in order to reveal the effect of the location of the metal and phantom within the rotational scan FOV. The used metal inserts were steel rods with a diameter of 3.2 mm. The rods were placed in the ilium bone mimicking structure next to a hip joint. Examples of the image artifacts produced by the bilateral metal inserts in the body phantom are presented in Fig. 1.

**Table 1:** Imaging protocol settings and corresponding simulated effective doses

Protocol name	kVp	mAs	mSv
Reference	100	100	1.2
Low tube voltage	80	100	0.6
High tube voltage	120	100	2.2
Low dose	100	40	0.5
High dose	100	400	5.0

An image volume from the hip area was reconstructed with a standard reconstruction algorithm of the device, producing slices of 0.833 mm thickness and a 21.25 cm × 21.25 cm display FOV with a matrix size of 512 × 512 pixels, resulting in a pixel size of 0.415 mm in the x- and y-directions. The pixel values of the image data produced by the CBCT scanner are presented in arbitrary contrast normalization instead of the calibrated Hounsfield unit range used in multi-slice computed tomography scanners.

### Image preprocessing

The image volumes with and without metal artifacts were first registered to take the slight rigid movement of the phantom (from the insertion and removal of the metal rods) into account. After registration, the artifact-free images were resliced without altering the original image volume resolution. All registrations were done with the 3D Slicer software [23].

After the registration procedure, a pixel-wise subtraction between the image volume with and without the metal artifact was calculated (Fig. 1). The lookup table (LUT) determining the presentation of image contrast was not similar between the image volumes, likely due to the corresponding differences in automatic pixel-value calibration of the preprocessed projection data. Thus, it was necessary to match the LUT of each image data set before the calculation of the subtracted image. However, linearity of the pixel values as a function of X-ray attenuation was presumed, despite a different LUT in each volume. The matching of the LUTs was done by measuring the mean intensity value of the bone material and background material of the phantom in a transverse image slice away from the metal insert. The subtraction image was then calculated with the following equation:

1 $$Subtraction\,volume=Artifact\,volume-\left(a\times Artifact{\text{-}}free\,volume-b\right).$$

Coefficients a and b were determined as

2 $$a= \frac{{I}_{B{O}_{A}}-{I}_{B{G}_{A}}}{{I}_{B{O}_{F}}-{I}_{B{G}_{F}}}$$

3 $$b= {I}_{BG_A }-a\times {I}_{BG_F}$$

where *I* is intensity, *BO*_*A*_ bone in the artifact image volume, *BG*_*A*_ background in the artifact volume, *BO*_*F*_ bone in the artifact-free volume, and *BG*_*F*_ background in the artifact-free volume. The quality of the subtraction was verified by looking at the pixel-value histogram of the subtracted image. With the correct subtraction, pixel values should be close to zero with the exception of values representing the artifact signal.

### Image analysis

**Fig. 2:**
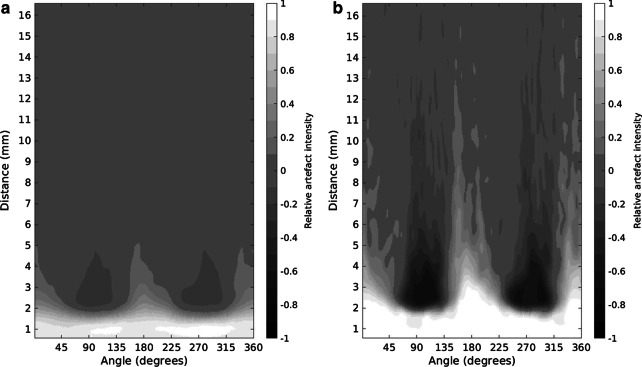
Relative pixel intensity value maps of metal artifact in the body volume with **a** 100 kVp and **b** 120 kVp tube voltages

A transverse image slice in the subtracted image volume was chosen where metal artifacts produced by the metal rods were clearly present. The image slice was first interpolated linearly by tenfold to increase the resolution in the calculation. The exact center of the metal artifact was located by searching the center of mass of the artifact contrast region. Pixel intensity values in the image were then recorded based on the distance and angle relative to the center point of the artifact. The used angle resolution was one degree with a three-degree moving window. The distance resolution was 0.1 pixel (0.0415 mm) with a one-pixel (0.415 mm) moving window. An example of a relative intensity value map with distance and angle is presented in Fig. 2. The size of the image artifact was studied by measuring how far from the center of the metal rod the intensity of the artifact signal had decreased to 25% of the maximum.

## Results

**Fig. 3.**
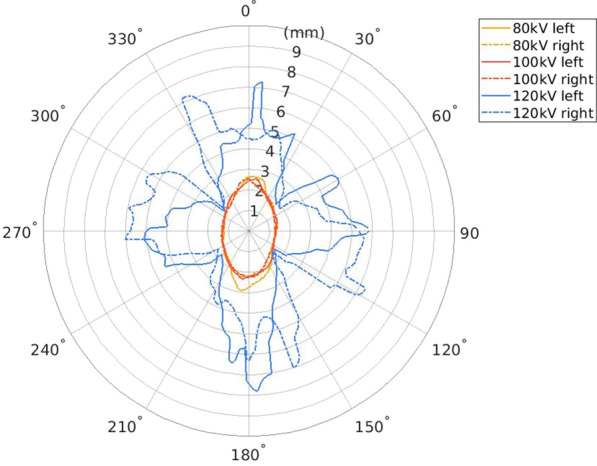
25% artifact isolevels of the maximum artifact intensity with respect to the used tube voltage measured as millimeters versus the angle from the artifact center

Figure 3 shows the 25% artifact attenuation isolevels with respect to the used kilovoltage and angle from the metal artifact center point in the axial plane. The artifact in the image volume obtained with 120 kVp was substantially larger than the corresponding artifact in the 80 kVp and 100 kVp image volumes.

**Fig. 4.**
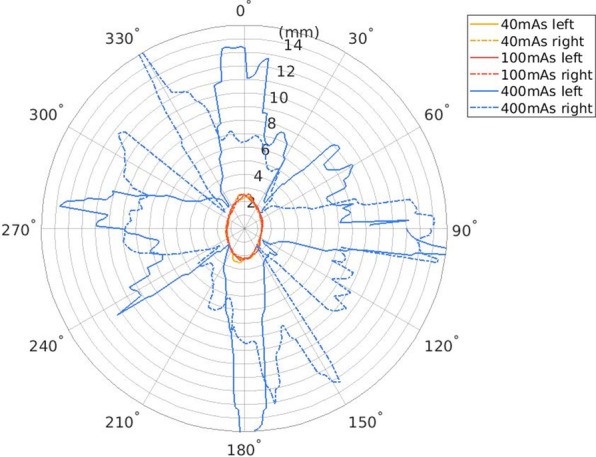
25% artifact isolevels of the maximum artifact intensity with respect to the used exposure measured as millimeters versus the angle from the artifact center

The 25% artifact attenuation isolevels with respect to the used mAs and angle from the artifact center in the axial plane are presented in Fig. 4. The artifact in the image volume obtained with 400 mAs was substantially larger than the artifact in the 100 mAs and 40 mAs image volumes.

The metal insert laterality or image volume isocenter position (i.e., off-centered phantom) did not have a significant effect on the artifact size with the used imaging parameters.

## Discussion

This study aimed to develop a robust method to quantify metal artifacts in CBCT and to apply this method to O-arm scans of an anthropomorphic phantom with metal inserts. When comparing the size of the metal artifact with the used x-ray tube voltage in an anthropomorphic pelvis phantom, the images acquired with 80 kVp and 100 kVp resulted in similar metal artifacts. However, with 120 kVp tube voltage, the resulting artifacts were considerably larger, which is counterintuitive with respect to the fundamental physics assumption of lowering contrast with higher photon energy. This nonlinear behavior of the imaging system with respect to the used kilovoltage could be explained by saturation of the detector signal with higher exposure levels reached with higher kilovoltage, possibly in connection with the automatic data preprocessing and image reconstruction of the O-arm system. Altogether, the size of the artifact with both 80 kVp and 100 kVp can be regarded as moderate in comparison with the metal rod diameter. With 120 kVp tube voltage, the total range of the artifact was greater but not extensive.

A similar effect, as with the different kVp settings, was observed in the phantom image volumes obtained with 40 mAs, 100 mAs, and 400 mAs. The artifact size obtained with 400 mAs was significantly larger in comparison with two smaller mAs settings. The artifacts in the 40 mAs and 100 mAs volumes were generally similar and small in comparison with the metal rod diameter. Strong nonlinear behavior regarding the mAs values further supports the assumption of detector signal saturation, potentially also reflecting the intensity scaling of the image data in the preprocessing. It should be noted that similar behavior regarding the metal artifacts with mA settings was also presented by Abul-Kasim et al. [13].

Regarding the basic physical photon interactions, it is expected that the attenuation differences in the soft and bone tissue density range contributing to the image contrast are larger with lower kilovoltages (such as 80–100 kVp). Higher kilovoltages (such as 120 kVp) will inevitably lead to lower contrast signal in the tissue contrast range. If the metal signal dynamics grossly dominate the signal irrespective of the applied kilovoltage, and raw-data projection image preprocessing does not compensate for this wide signal difference range, this may lead to observed accentuated artifacts. This phenomenon is clearly visible in the applied CBCT system. However, it may not be generalizable to other CBCT systems.

The image volume isocenter and metal insert laterality did not have a significant effect on the artifact size. The metal inserts were located far away from each other compared with their size, which mostly excluded their mutual interference. Based on the results, the applied O-arm image reconstruction also seemed relatively tolerant to out-of-field (truncation) image artifacts.

The tendency of the imaging system to change the slope of the LUT in the images produces additional uncertainty in the results in terms of the presented contrast scale. The system seemed especially prone to alter the LUT when a metal insert with high attenuation was included in the image volume. In our method, it was presumed that the linearity of the LUT is not changed in the process. This assumption was verified by analyzing the intensity value histogram of the subtracted images after the fitting of the LUTs.

## Conclusions

The presented method offers a robust way to quantify metal artifacts in CBCT images. The quantification of metal artifacts is presented for the O-arm scans of an anthropomorphic phantom with metal inserts. Changes in protocol parameters may have nonlinear effects on the image quality which are not explained by physics and represent the vendor-specific processing of the acquired image data.

## Data Availability

The data sets used and/or analyzed during the current study are available from the corresponding author on reasonable request.

## Abbreviations

3D: three-dimensional
CBCT: cone-beam computed tomography
FOV: field of view
LUT: lookup table

## References

[1] Holly LT, Foley KT (2009). Three-dimensional fluoroscopy-guided percutaneous thoracolumbar pedicle screw placement. J Neurosurg.

[2] Acosta FL, Thompson TL, Campbell S, Weinstein PR, Ames CP (2005). Use of intraoperative isocentric C-arm 3D fluoroscopy for sextant percutaneous pedicle screw placement: case report and review of the literature. Spine J.

[3] Bledsoe JM, Fenton D, Fogelson JL, Nottmeier EW (2009). Accuracy of upper thoracic pedicle screw placement using three-dimensional image guidance. Spine J.

[4] Nottmeier EW, Pirris SM, Edwards S, Kimes S, Bowman C, Nelson KL (2013). Operating room radiation exposure in cone beam computed tomography-based, image-guided spinal surgery. J Neurosurg.

[5] Mason A, Paulsen R, Babuska JM (2014). The accuracy of pedicle screw placement using intraoperative image guidance systems: a systematic review. J Neurosurg.

[6] Kleck CJ, Cullilmore I, LaFleur M (2016). A new 3-dimensional method for measuring precision in surgical navigation and methods to optimize navigation accuracy. Eur Spine J.

[7] Dea N, Fisher CG, Batke J (2016). Economic evaluation comparing intraoperative cone beam CT-based navigation and conventional fluoroscopy for the placement of spinal pedicle screws: a patient-level data cost-effectiveness analysis. Spine J.

[8] Rivkin MA, Yocom SS (2014). Thoracolumbar instrumentation with CT-guided navigation (O-arm) in 270 consecutive patients: accuracy rates and lessons learned. Neurosurg Focus.

[9] Ryang YM, Villard J, Obermuller T (2015). Learning curve of 3D fluoroscopy image-guided pedicle screw placement in the thoracolumbar spine. Spine J.

[10] Costa F, Tosi G, Attuat L (2016). Radiation exposure in spine surgery using an image-guided system based on intraoperative cone-beam computed tomography: analysis of 107 consecutive cases. J Neurosurg.

[11] Su AW, Luo TD, McIntosh AL (2016). Switching to a pediatric dose O-arm protocol in spine surgery significantly reduced patient radiation exposure. J Pediatr Orthop.

[12] Su AW, McIntosh AL, Schueler BA (2017). How does patient radiation exposure compare with low-dose o-arm versus fluoroscopy for pedicle screw placement in idiopathic scoliosis?. J Pediatr Orthops.

[13] Abul-Kasim K, Söderberg M, Selariu E, Gunnarsson M, Kherad M, Ohlin A (2012). Optimization of radiation exposure and image quality of the cone-beam O-arm intraoperative imaging system in spinal surgery. J Spinal Disord Tech.

[14] Schouten R, Lee R, Boyd M (2012). Intra-operative cone-beam CT (O-arm) and stereotactic navigation in acute spinal trauma surgery. J Clin Neurosci.

[15] Qureshi S, Lu Y, McAnany S, Baird E (2014). Three-dimensional intraoperative imaging modalities in orthopaedic surgery: a narrative review. J Am Acad Orthop Surg.

[16] Riis J, Lehman RR, Perera RA (2017). A retrospective comparison of intraoperative CT and fluoroscopy evaluating radiation exposure in posterior spinal fusions for scoliosis. Patient Saf Surg.

[17] Boas FE, Fleischmann D (2012). CT artifacts: causes and reduction techniques. Imaging Med.

[18] Tang X, Krupinski EA, Xie H, Stillman AE (2018). On the data acquisition, image reconstruction, cone beam artifacts and their suppression in axial MDCT and CBCT–A review. Med Phys.

[19] Katisko JP, Kauppinen MT, Koivukangas JP, Heikkinen ER (2012). Stereotactic operations using the o-arm. Stereotact Funct Neurosurg.

[20] Sharma M, Deogaonkar M (2016). Accuracy and safety of targeting using intraoperative “O-arm” during placement of deep brain stimulation electrodes without electrophysiological recordings. J Clin Neurosc.

[21] International Atomic Energy Agency, Status of Computed Tomography Dosimetry for Wide Cone Beam Scanners, Human Health Reports No. 5, IAEA, Vienna, 2011. 39 pages, link: https://www.iaea.org/publications/8758/status-of-computed-tomography-dosimetry-for-wide-cone-beam-scanners. Accessed 20 Nov 2020

[22] International Electrotechnical Commission, Medical electrical equipment - Part 2–44: Particular requirements for the basic safety and essential performance of X-ray equipment for computed tomography, IEC 60601—2-–44:2009+AMD1:2012+AMD2:2016 CSV Consolidated version, Edition 3.2, IEC, Geneva, Switzerland, 2016, 276 pages

[23] Pieper S, Halle M, Kikinis R. 3D Slicer. Proceedings from the 2004 2nd IEEE International Symposium on Biomedical Imaging. Macro to. NANO. 2004;2:632–5.

